# Struggles for provision of palliative care services at the primary healthcare setting in Tanzania; Experiences from health facility in-charges in Dar es Salaam; A qualitative study

**DOI:** 10.1371/journal.pone.0342485

**Published:** 2026-02-12

**Authors:** Christina Vallen Malichewe, Furahini Yoram, Juda Lyamai, Veronica Mkusa, George Kiwango, Obadia Nyongole, Nathanael Sirili

**Affiliations:** 1 Department of Clinical Oncology, Muhimbili University of Health and Allied Sciences, Dar es Salaam, Tanzania; 2 Department of Research and Empowerment, Ant-Poverty Sensitization and Community Development Planning Foundation, Lindi, Tanzania; 3 Palliative Care Trainers and Researchers Network Tanzania, Dar es Salaam, Tanzania; 4 Department of Human Physiology, Muhimbili University of Health and Allied Sciences, Dar es Salaam, Tanzania; 5 Department of Surgery, Muhimbili University of Health and Allied Sciences, Dar es Salaam, Tanzania; 6 Department of Development Studies, Muhimbili University of Health and Allied Sciences, Dar es Salaam, Tanzania; Epicentre, FRANCE

## Abstract

**Background:**

The rising burden of chronic illnesses due to Communicable Diseases and Non-communicable Diseases has heightened the need for palliative care services globally. The World Health Organisation (WHO) advocates for palliative care integration from the lower levels of the healthcare system. However, health systems in many low—and middle-income countries suffer from a lack of palliative care services at the lower level.

This study aimed to explore the perceptions of the hospital in charge (the managers from 15 Health Centre facilities) on available healthcare services that can facilitate the integration of palliative care services at primary care facilities in Dar es Salaam, Tanzania.

**Methods and findings:**

An exploratory qualitative case study on the perceptions of purposefully selected health facilities in charge of the available healthcare services, which can be used to facilitate the integration of palliative care services in primary care facilities. A semi-structured interview guide was used to conduct in-depth interviews with the selected informants. Data was analysed using a content analysis approach. Two categories emerged from the in-depth interviews: the availability of physical support services to manage pain and symptoms and multiple support systems to facilitate care for patients with chronic illnesses. Health facilities in charge consider the availability of various healthcare services at primary healthcare facilities essential for integrating palliative care services at this level. However, these services do not work in the context of palliative care and lack the philosophy behind it.

**Conclusion:**

The availability of these services is a starting point for developing palliative care philosophy and, thus, its integration. Capacity building on palliative care services at the Primary health care facilities is recommended.

## Introduction

Palliative care, a holistic approach to patients with life-threatening illnesses, is increasingly integrated into primary healthcare (PHC) settings, focusing on early diagnosis, treatment, and rehabilitation to improve patients’ quality of life and their families [1,2]. Primary health care is critical to palliative care services because of its comprehensiveness, patient-centered, and community-based approach [3]. It guarantees patients receive all the care they need, including attention to their emotional, social, and spiritual needs as well as their medical symptoms, with an emphasis on accessibility, coordination, and continuity of care [4].

Patients with chronic illness suffer from a wide range of pain and symptoms related to the chronic disease they are suffering from [5]. Primary healthcare facilities need palliative care services, for patients focusing on wholeness, physical, psychosocial, cultural, and spiritual well-being, and incorporating a multidisciplinary approach involving various stakeholders in care [6]. It is reported that damage to one aspect of a person, for example, having physical pain, can lead to emotional distress that can affect the total functioning of an individual, leading to poor psychological and social performance [7].

The experiences of delivering palliative care services at primary healthcare facilities present both rewards and challenges. It is rewarding because it facilitates collaborative efforts among primary healthcare professionals, social workers, chaplains, and psychologists [8]. Continuous education and training programs equip PHC service providers with quality palliative care service delivery skills and provide patient-centered care [9]. Conversely, the challenges are attributed to limited resources (skilled service providers, medical equipment, and medications), emotional burdens [10], and communication barriers [11]. The collaboration between policymakers, healthcare organisations, and educators is essential for PHC facilities to enhance the quality of life for palliative patients by providing enough funding, staffing, medication, and equipment [12].

Palliative care in Tanzania is still relatively new, especially in PHC facilities. Therefore, it is worth understanding PHC facilities’ medical officer-in-charge perceptions regarding the holistic approach to caring for patients with chronic illness, encompassing the physical, social, psychological, financial, and spiritual support and other factors that facilitate the integration of palliative care into their workplace, since they oversee the healthcare delivery at the health facility. This study aimed to explore the perceptions of the hospital in charge (the managers from 15 Health Centre facilities) on available healthcare services that can facilitate integrating palliative care services at primary care facilities in Dar es Salaam, Tanzania. The person in charge of a primary health facility in Tanzania oversees daily operations, ensuring efficient service delivery (maternal-child health, immunization, and outpatient services), supervision and mentorship of staff, managing drug supplies, providing health data reporting to district authorities, handling administrative work, as well as maintaining the quality of the services [13].

## Materials and methods

### Study design

The study used a qualitative case study design, with semi-structured interviews with primary health care facility in-charges in Dar es Salaam, Tanzania, to explore the perceptions of the hospital in charge (the managers from 15 Health Centre) facilities) on available healthcare services that can facilitate the integration of Palliative care services at primary care facilities in Dar es Salaam, Tanzania. The design allows for an in-depth exploration of the perceptions of those in charge in their work context.

### Study context

A decentralized system is used in the United Republic of Tanzania to provide health care [14]. Dispensaries, health centers, and district hospitals are all part of the President’s Office, Regional Administration, and Local Government, which is in charge of providing services. In general, the decentralized health system can be divided into three functional levels: referral hospitals (tertiary level), districts (primary level), and regions (secondary level). The primary level is the main context of our study. The district is tasked with organizing, carrying out, overseeing, and assessing health services. PHC services are delivered at the district level via ward-level dispensaries, accommodating three to five villages. The health center, a referral level for the dispensary, provides a wide range of services. Additionally, a district hospital serves as a referral level for health centers. The study purposively selected 15 public and private primary healthcare facilities from a total of 322 hospitals, health centers, and dispensaries found in the area.

### Sampling strategy

Purposive sampling was used to select 15 Health Centre facilities to collect primary data on the available services for integrating palliative care. In each PHC facility, the health facility in charge was interviewed. To allow a full range of perspectives among health care facility in-charges, the participants were purposively recruited from the facilities with high population density, location (urban or semiurban) and public and private owned facilities [13]. The inclusion criteria included high-patient-density facilities and facilities managed by health professionals. In 15 PHC facilities, ten hospital in-charges were female, and five were male. The participants’ ages ranged from thirty to seventy-three years. The participant’s cadre, education level, and facility type are described in Table 1. The data saturation determined the required participants. This study assessed saturation using concurrent data collection and analysis [15]. The researchers determined saturation when no new themes emerged after three consecutive interviews [16]. The researchers conducted ongoing assessments of themes through weekly coding meetings and refinement of the codebook. To enhance reliability, three researchers reviewed the thematic saturation.

**Table 1:** The characteristics of the participants interviewed.

Participant cadre	Education level	Facility type
Clinical officer	Diploma	Public
Medical Doctor	Degree	Public
Nurse	Diploma	Private
Medical Doctor	Degree	Private
Medical Doctor	Degree	Public
Clinical officer	Diploma	Public
Clinical officer	Diploma	Public
Specialist	Masters in Medicine	Private
Clinical officer	Diploma	Public
Clinical officer	Diploma	Public
Specialist	Masters in Medicine	Public
Clinical officer	Diploma	Private
Specialist	Masters in Medicine	Private

### Data collection

A letter for an interview appointment and a schedule were sent to the health facility in charge so they could choose a convenient date to participate. Before the interviews, the research team established rapport with the participants by reading an information sheet that outlined key study details, including the conducting institution, research objectives, and goals.

Then the informed consent form was signed. Then, interviews were conducted face-to-face in the office of the health facility in charge from August 11^th^ −18^th^, 2023. The interviewer used a tape recorder and a notebook to record non-verbal gestures; only one person conducted one interview. Every day, after interviews, the research team checked the audio files for completeness and an assessed new concept that emerged. The audio files were uploaded to the computer folder, and transcription was done.

An interview guide was used to gather information. The guide was piloted and edited. The guide had questions that explored the services offered at PHC facilities and how those services were organized. These questions were followed by probe questions based on participants’ responses. During In-depth interviews, the Kiswahili language was used, as it is the language most participants speak fluently. Two research assistants conducted the interviews: 1 Female and one male. Experienced in conducting qualitative interviews. The interviews lasted 20–45 minutes. However, the research assistants were trained before the data collection activity. The research assistant credentials: the female was a PhD student and the male had a Master’s degree. The research assistants had no specific interest in the study, so there was no bias. None of the authors collected data; two research team members supervised the interviewers.

### Data analysis

The researchers transcribed the audio-recorded in-depth interviews verbatim. The analysis was done manually in Kiswahili to maintain the original meaning. Later, codes, subcategories, categories, and quotes were translated from Kiswahili to English. Inductive qualitative content analysis guided the analysis [17]. From the reduced-meaning unit, codes were isolated. Based on their similarities and differences, codes were grouped into subcategories, leading to the emergence of the categories. The whole process was inductive and iterative, and the team of three agreed on codes and categories using quotes from the participants. The coding was validated through the intercoder reliability, using a percentage agreement method. The inter-coder agreement was reported to be 85%, indicating high consistency in coding [18].

#### Reflexivity.

The authors’ previous training influenced the understanding and explanation of the data, possibly highlighting gaps in service delivery. Professionals in qualitative data collection were employed to gather information and reduce bias during the interviews, but the analysis may reflect perceptions of ideal palliative care. To alleviate this, the authors maintained a reflective journal and critically observed their assumptions during the study to ensure sensible results [19].

#### Trustworthiness.

To ensure the credibility of the data, we involved researchers from different backgrounds and selected primary care facilities from public and private settings. Deriving the categories from study participants’ experiences working in PHC facilities, rather than from the researchers’ interpretations improved conformability. Researchers performed the interviews at each study participant’s location to guarantee reliability. To enhance dependability, researchers maintained the details of the interview guide, field records, and coding processes. By providing a thorough explanation of the process, we improved transferability.

### Ethical consideration

Ethical clearance for the study was obtained from the Institutional Review Board of the Muhimbili University of Health and Allied Sciences (Ref. No.DA.282/298/01.C/1763). Permission to conduct this study was acquired from the President’s Office, Regional Administration and Local Government Tanzania, the Dar es Salaam, Regional Secretariat, the City Council Authority, and the hospital management teams. After the detailed explanation of the study, written informed consent was obtained from each participant. Participants were informed of their right to refuse or withdraw from the study. All the interviews were conducted in a well-prepared room to keep participants’ privacy. Also, participants’ records were kept confidential.

## Results

**Fig 1:**
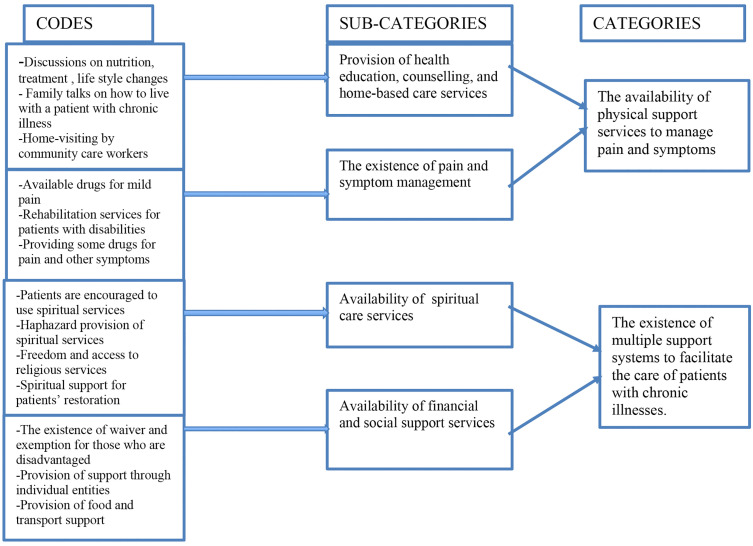
Summary of interview findings.

Two categories emerged from in-depth interviews with managers of primary care facilities about their perceptions of resources available to facilitate the integration of palliative care services at their facilities. These are 1)the physical support services available to manage pain and symptoms and 2) multiple support systems available to facilitate the care of the patients (Fig 1).

### Category 1: Available physical support services for pain and symptom management

In this category, two subcategories emerged—:the provision of health education and home-based care services, and the existence of pain and symptom management systems.

#### Provision of health education, counselling, and home-based care services.

PHC facilities offer health education on lifestyle changes and psychosocial support when serving patients with chronic illnesses. Health education is related to diet, lifestyle, and adherence to medications. One health facility in charge narrates:

*“[…] I tell them to change their lifestyle. Maybe they should eat less salt, exercise, take […] medicine on time, go to the hospital, and reduce fat. [For] diseases like mental illness, we tell them not to stay near dangerous environments such as fire, motor cars, drugs, so there should be someone to help them. For AIDS patients […], we help them manage those opportunistic diseases. So, we advise them to eat on time, drink water, and have time to rest [Health Facility (HF) in-charge-07]*

The quote echoes a holistic approach to palliative care, advocating for health providers to manage symptoms and provide health education on lifestyle guidance, safety actions, and psychosocial support to address complicated pain, psychological distress, and progression to advanced disease.

Several participants brought up a lack of psychological assistance at PHC facilities. Although some counselling services available, they are sometimes short-lived and have a narrow scope, necessitating referrals to other clinics. As one facility in charge clarifies

*“We have people come in with stress, but we do not have specific management. What we do is counsel them for the short time we have, and then we refer them to respective clinics/facilities” (HF in-charge - 04).*

The quote stresses how important it is to incorporate palliative care education within PHC services, especially when it comes to treating psychosocial distress.

The availability of Community health workers (CHWs) visiting patients’ houses and regularly providing health education to patients and caregivers was highlighted by the participants. CHWs can be a model for many patients to access palliative care at home; the CHWs act as an essential link between the health facility and the community, as one in charge explains:

*“We have community health workers who provide home-based health services and education at the household level,. Therefore, if a patient needs home care, we connect them with these providers — they are based here at the facility and are assigned to different neighborhoods. We also collaborate with local government leaders, which helps us to know where each patient lives and how to reach them”. (HF in-charge - 01)*

The above quote highlights the significant potential role of community health workers’ in facilitating access to palliative care in Tanzania. The creation of a network of families with CHWs at the community level guarantees the provision of home-based palliative care services. The distributed CHW networks support early recognition of palliative desires and ensures stability of care, as recommended by WHO for community-integrated palliative care [20].

#### Existence of pain and symptom management services.

Most participants explained they provide pain and symptoms management at PHC facilities, offer pain relief drugs, and treat symptoms such as gastric pain or injuries. PHC facilities in charge reported the lack of some essential medicines for pain management. Participants report managing severe pain using Paracetamol or Diclofenac injections, which are drugs for the treatment of mild pain. One participant narrates:

*“Regarding pain management, it depends on the history [from the patient]. We use diclofenac injection although others are allergic to it, so we use Paracetamol IV and Tramadol if paracetamol are out of stock”. (HF in-charge – 04).*

The quote shows that there are critical gaps in the management of pain in the palliative care context at the primary health care level. The health care providers are required to tailor pain treatment based on the patients’ needs. The dependence on paracetamol, diclofenac and limited access to opioids (Morphine) reveals the inadequate access to essential severe pain management drugs.

Participants mentioned the inability to obtain necessary painkillers, especially opioids like morphine, as a significant obstacle. Obtaining ongoing access at PHC facilities was frequently difficult for patients who had received oral morphine prescriptions at referral hospitals. One of the primary health center’s facilities in charge explains:

*“… we usually receive patients who claim to be palliative care patients; according to them, they come with their treatment either from Ocean Road or Muhimbili National Hospital. They come with their oral morphine, which is not available in our facility”. [HF in-charge-15]*

The quote demonstrates the substantial supply chain and policy obstacles that stand in the way of Primary health care facilities providing appropriate pain management.

Challenges in the availability of drugs for the provision of pain and symptom management significantly shaped palliative care delivery at primary health care facilities. As one of the health facilities in charge, states:

*“There are some medications that we are not authorized to prescribe, but we do help relieve pain as much as possible— for example giving medication to help the patient sleep and rest better. [HF in-charge-1]*

The quote shows three essential issues: the restrictions of controlled formularies, improvisations with existing analgesics, and the emphasis on symptomatic relief when the ideal treatment/drugs/medications are not available.

### Category 2: The existence of multiple support systems that facilitate care for patients with chronic illness at PHC

There are multiple support systems available for patients with severe illnesses at PHC. The category evolved from two subcategories, including the availability of spiritual support services and the provision of financial and social support services.

#### Spiritual support services that enhance the provision of palliative care services.

Participants stressed the need for spiritual support in palliative care. While PHC facilities do not have professional spiritual counselors, they regularly connect patients with religious leaders according to their beliefs. As one in charge explains:

*“We link [patients with serious illness] with their religious affiliations according to their religious beliefs. Christians are linked to the Pastors or whoever. Others have been receiving the last spiritual services. This work is being done by the social welfare officers” (HF in-charge - 06).*

The psychological advantages of spiritual care were emphasized by another participant, who notes:

*“Spiritual care is a treatment, it is psychological assurance, psychological treatment, for example, let me say I am very sick [and] spiritual people came and prayed for me, then I felt as if I was cured, you see, so it has psychological treatment as well. [This comes] because many people are affected psychologically, especially for these [life] threatening diseases, so they need spiritual counselling.” (HF in-charge-14)*

These answers imply that although spirituality is acknowledged as an essential element of holistic treatment, PHC facilities do not have systematic methods for incorporating it into their services.

Another participant recognized the need for skills in assessing and communicating with patients and families who have spiritual needs. It is not easy to have spiritual conversations with patients unless you are trained to do so, which most healthcare providers are not. One participant explains:

*“I think spiritual care is an important area. It would be helpful if we could have someone responsible for that — it would help patients live with hope because faith plays a big role in healing and comfort.”. [HF in-charge - 9]*

The quote shows that there is a need for training health care providers in PHC facilities on handling spiritual issues in palliative care settings to improve the quality of life for patients with chronic illness.

#### Availability of financial support.

Participants reported that PHC facilities provide waivers and exemptions to disadvantaged groups, including patients with serious illnesses. Underprivileged groups include people with disabilities, emergencies such as accident victims, and those who are economically poor regardless of their age. Further, a few participants mentioned that PHC facilities through social welfare offices provide financial and material support to vulnerable groups, such as older people, to address various social needs occasionally. One of the health facilities in charge describes:

*“We have economically poor clients (both elderly and non-elderly) who will tell you, look, the doctor prescribed this medicine for me, but I cannot afford it. We exempt people of this kind. Some clients are exempted from medicines, other people with disability are given wheelchairs if available*” *(HF in-charge - 05).*

In addition, the participant acknowledges that one of the social workers’ responsibilities is facilitating financial relief for the patients. One participant state:

*“…social worker, we know one of her duties is to provide an exemption to patients who present to their office; this is one of the palliative care services”. [HF in-charge-8]*

These quotes imply the need for a compassionate and patient-centred approach in implementing holistic care, such as addressing financial and physical barriers that patients encounter to safeguard equal access to basic palliative care services. Also, contribution of social workers in the holistic care is significant.

#### Availability of social support.

The participants reported the systemic gap in the availability of social support services with in the health care facilities. One participant note:

*“Currently, we have no social support services from our facility. Instead, we link patients who need social support to religious institutions that provide this kind of support”. [HF in-charge-6]*

This reply highlights the dependence on peripheral institutions, including religious groups, to fill the gaps in psychosocial support for palliative care clients. There is a need to strengthen integrated social support within healthcare systems to ensure comprehensive, compassionate palliative care for all.

Primary healthcare facilities rely on referrals of critically ill patients for specialized care to higher facilities in the healthcare system. One of the healthy facilities in charge reports:

*“ If we see that the patient needs more advanced medical support, we give them a referral to Amana Hospital. As a facility, we can help facilitate transport or sometimes request an ambulance to transfer them.” [HF in-charge-13]*

The quote emphasizes the significance of the referral systems and logistical support in caring for patients with palliative care needs. Some facilities help in alleviate the access barriers through by arranging transport.

## Discussion

This study aimed to explore the perceptions of the hospital in charge (the managers from 15 health center facilities) on available healthcare services that can facilitate the integration of Palliative care services at primary care facilities in Dar es Salaam, Tanzania. Participants in our research reported providing a wide range of services for patients with chronic illnesses, including counselling, health education, home-based care from community health workers, pain and symptom management, spiritual support, and social and financial assistance.

In our study, participants reported that primary care facilities deliver health education to patients with chronic illnesses, focusing on diet, lifestyle, and medication adherence. Health education is a core function of primary healthcare [21], but our findings suggest that current efforts lack a palliative care component. While participants acknowledged offering lifestyle advice, the absence of end-of-life discussions indicates a gap in holistic care approaches.

The results align with research by Moreno-Parel et al. [22] and NNakakuwa et al. [23], who found that health promotion in PHC settings often prioritizes disease prevention over symptom management in chronic illness. Given that chronic illnesses frequently progress to life-threatening stages, integrating palliative care education into routine health promotion could improve patient outcomes. Further research is needed to assess the impact of palliative care-focused health education in PHC settings.

Our study reports a lack of psychological assistance for patients experiencing stress. There is an essential gap in mental health support in primary healthcare settings, especially on the issues of stress management, which is an everyday complaint. However, it is a frequently underestimated feature of palliative care. The brief counselling services provided at the primary health facility, followed by a referral, highlights limitations in the health system regarding time, funds, assets, and training. These services focus on addressing psychosocial distress, a vital component of holistic palliative care as recommended by WHO [4]. In research by Swathi M et al. [24], psychological conditions such as anxiety, stress/tension, and depression often increase with chronic or life-limiting illnesses, aggravating physical symptoms and lessening the quality of life. Identifying stress as the main problem, along with the absence of coordinated interventions, highlights a missed opportunity for prompt, combined psychosocial care—a foundation of successful palliative practice, as explained by Radbruch et al. [25] in their study on redefining palliative care, which offers a new consensus-based definition.

The brief counselling and the reliance on referral to specialized clinics uncover health provider pressures (limited time, lack of training) and disintegrated care systems whereby mental health is stand-alone rather than intertwined into regular palliative services [4]. The result supports the global call for task-shifting approaches to train primary healthcare workers with basic psychosocial skills ensuring appropriate support without overstraining the referral systems [20]. Therefore, it is necessary to integrate mental health screening into palliative care protocols, supported by health system investments in training, human resources, and combined care models to widely tackle this unmet need.

Community Health Workers play a role in bridging gaps in palliative care delivery, especially in underserved populations. In this study, CHWs reported providing home-based health services and education, ensuring that palliative care extends to needy families, despite facing barriers such as limited health access and social and financial disparities. Their positions within the communities permit culturally directed care, building trust, and refining patient and family involvement in end-of-life decision-making [26]. Connecting CHWs to specific streets reported in our study warrants localized and uninterrupted care, which is vital in palliative care because follow-up and symptom management are critical [27]. Additionally, CHWs act as the intermediates between health facilities and families, assisting in the early identification of palliative care needs and early referrals [26]. Therefore, there is a need for a structured system to manage the CHWs ensuring they provide their services effectively in the palliative care context. Integrating CHWs into palliative care in the primary care system is a model that ensures early identification of people in need, management of symptoms, provision of psychosocial support, and facilitation of community engagement [26–28]. However, a review by Herber & Johnstone [29] reports some challenges that prevent the CHWs from effectively functioning, including insufficient training, emotional burden, and scarcity of resources. We can strengthen the CHWs’ programs by developing structured training with a palliative care context, integrating them into primary healthcare system, and supervising activities in their communities which are essential for long-term palliative care provision. The CHWs model demonstrates how community-based approaches can facilitate equity in palliative care, ensuring all in need receive dignified end-of-life care.

Our study revealed that a patient-centered approach is an effective way of managing pain in a palliative care context, which requires individuals’ health histories, reactions to drugs and availability of medication. The use of first-line pain drugs such as diclofenac injections in managing severe pain, with alternatives including intravenous paracetamol or tramadol, mirrors the adaptive approaches needed in resource-constrained environments. The intravenous route is faster, reliable and convenient for patients with severe pain, since it bypasses the gastrointestinal tract and first-pass metabolism in the liver [30]. Studies have shown that a combination of diclofenac injection and paracetamol infusion was better in controlling pain with few side effects in the management of postoperative pain [31,32] that required opioids. However, a review on non-steroidal anti-inflammatory drugs (NSAIDs) and pain in cancer patients shows the lack of the finest evidence about the analgesic effectiveness of NSAIDs in chronic cancer pain [33], as well as the increased risk of liver toxicity with paracetamol if used for chronic pain management [34]. Showing the need for the availability and accessibility of opioids in cases of patients with chronic pain, such as cancer. Furthermore, the use of injectables is not without side effects and undesirable effects [35,36]. Therefore, the choice of pain treatment modality should be individualized.

The World Health Organisation recommends on the use of the oral morphine, for severe pain in patients with chronic illnesses [4]. However, its accessibility remains a problem in various low-resource settings [37]. The use of tramadol, a weak opioid, as a substitute in this study underscores the systemic gaps in the availability of potent opioids, which is serious for successful palliative pain control. The results are in line with a study from Taiwan in which the authors investigated the increasing use of tramadol under strict control of opioid usage, which revealed that the use of tramadol increased by 92.2-fold in patients without cancer [38]. In Tanzania, there is a need for comprehensive pain control guidelines that could increase the availability and accessibility of oral morphine and adjuvant analgesics [39].

In this study, we have highlighted a critical gap in palliative care delivery: the inequalities in distributing necessary drugs such as oral morphine throughout the healthcare facilities. A cancer patient who received oral morphine from tertiary centers (e.g., Ocean Road or Muhimbili National Hospital) presents with prescribed morphine to a local health care facility. Yet, local primary healthcare facilities lack this essential medicine, echoing systemic disparities in access to pain management drugs. A thesis report by Hofmeyr G [40] from South Africa on “an analysis of oral morphine use at primary healthcare facilities in the Cape Town Metro health district” aligned with our study, which reported that oral morphine prescriptions were more in the tertiary hospitals than in district and primary care facilities [41]. This scenario of the lack of morphine in primary health facilities reported in this study underlines the challenges of decentralized palliative care in resource-limited settings, which was also reported by Hofmeyr G [40], where dependence on higher-level and specialized hospitals for opioids interrupts the continuity of care and causes an unnecessary burden on patients to obtain medications [41].

Regulatory barriers exist that hinders the access of morphine, such as the limited number of authorized prescribers. In Tanzania, only doctors are allowed to prescribe morphine, whereas in Uganda, nurses have been trained and involved in pain management [42]. Supply chain inadequacies and a lack of training for healthcare providers contribute to scarcities in peripheral facilities, as reported in a study in Ethiopia by Fentie & Selam [43]. This disintegration threatens treatment interruptions, worsening suffering for patients who need to travel long distances to restock their treatments. The accessibility of opioids needs to be strengthened at all healthcare levels in Tanzania—via policy modifications, health provider education, and integrated supply schemes—and is critical to align with the principle of equitable palliative care [39]. Deprived of such actions, discrepancies in pain relief will continue, excessively distressing populations with chronic illnesses in underserved areas.

Patients, caregivers, and families often seek spiritual services. PHC facilities allow religious leaders to provide spiritual services for inpatient clients and encourage belief in God. The findings were also confirmed by McInnerney D et al. [44], who found that spiritual matters provide communication and emotional support to patients and their families. It encourages open and empathetic discussion that can answer and alleviate distress about the meaning of life, death, and suffering. Religious leaders often have spiritual influence over chronically ill patients and their families [45]. They can engage patients in a discussion about their lives, beliefs, values, and goals. A solid spiritual foundation means a person experiences less pain and distress, which enhances resilience and helps families and caregivers cope with grief and loss [46]. However, this service is not structured in our study area, as healthcare providers offer the service in an uncoordinated manner. We recommend that healthcare facilities assess the need for spiritual services and develop a structured referral system to channel patients to spiritual care providers according to the patient’s faith. Train healthcare providers and community health workers to identify and address patients’ spiritual needs and refer them appropriately. The healthcare facilities should collaborate with local faith-based organisations and community groups in their area to provide comprehensive spiritual welfare to their patients.

Some chronically ill patients, older people, disabled people, and low-income families are excluded from paying for services received from the healthcare facilities. The effectiveness of providing exemptions to older people to access health services is explained by Ntahosanzwe & Rwegoshora [47]. In their study, 323 participants were involved; 302 were older adults. The study found that the exemption policy is implemented but is less effective. Barriers to its implementation include the absence of necessary medications, rigid for exemption processes, and informal payments. Choosing which patients to exempt is a complex procedure [48]. However, the exemption is intended to safeguard children under five, expectant women, elderly patients, people with impairments, and people with chronic illnesses like cancer, diabetes, leprosy, Tuberculosis, polio, and HIV/AIDS [49–51]. Individuals’ income, health, behaviour, nutritional condition, and other factors influence their eligibility for subsidized health care. Some of the groups of people mentioned have life-limiting or life-threatening illnesses, which is the focus for palliative care [4]. Knowledge of palliative care will help healthcare providers identify patients who need exemption. Therefore, integrating palliative care in health care facilities will enhance the decisions on which patients really need to be exempted, improving the quality of life of patients and their relatives.

Our study revealed a critical gap in the provision of holistic palliative care, where there is a lack of institutional social support services necessitating reliance on religious groups to meet patients’ psychosocial desires. Integrating social services within primary healthcare facilities meaningfully increases the quality of life for patients and families by addressing practical needs, financial issues, and emotional challenges. A scoping review on *“Social workers’ coordination in primary healthcare for patients with complex needs”* revealed that social workers can coordinate various forms of social services, linking with activities that link patients, families, and health professionals to improve the fragmentation of care and achieve better health outcomes [52]. The existing model describes the risks of disintegrating care and may fail to meet criteria set by the WHO, which highlights multidisciplinary palliative teams, including social workers, therapists, and public support [20]. The palliative care model can strengthen primary health facilities by developing recognized collaborations with social services while creating competence for basic psychosocial support among clinical staff in low-income countries [53]. Policy amendments must prioritize funding for social care sections to ensure all patients obtain person-centered palliative support, irrespective of religious connections.

Our study reveals strengths and restrictions in the existing referral system. While providing transport services to higher-level care proves credible, it struggles to overcome access difficulties [54], which may unintentionally disrupt the delivery of palliative care. Repeated referrals can disturb the continuity of care, which is challenging primarily for patients with advanced sickness who benefit best from steady, longitudinal care interactions. However, a review by Ghanbari-Jahromi et al. [55] reported patient factors, such as disease-related, psychosocial, and individual factors along with institutional factors, including health system infrastructure and financial issues, contribute to the disruption of continuity of care. The importance of hospital-based “higher medical services” lies in their disease-oriented approach, which contrasts with complete palliative care that could be provided locally with appropriate support, such as community palliative care, potentially reducing the rate of referrals [56]. Studies reveal that successful palliative care integration needs the establishment of community-based services to lessen unwanted hospital transfers, which can be physically and emotionally demanding for seriously ill patients [56,57]. The transport provision informs a practical barrier [54]. An ideal system would integrate this with capacity-building at primary care levels to accomplish most palliative needs locally, reserving referrals for truly complex cases. Transport services help carers who lack the resources to transport their patients to a higher health facility, to focus on emotional support and companionship. We urge healthcare facilities to allocate a budget for patient referral services to alleviate the burden on patients and families during the challenging care process.

## Conclusion and recommendation

The available services provided at the primary care level can facilitate the integration of palliative care for patients with life-threatening illnesses and their caregivers. The gap observed is that the services delivered lack the knowledge and philosophy of palliative care. To address the gap and respond to the ever-increasing demand for palliative care services, it is necessary to raise awareness through advocacy and train healthcare professionals to provide quality healthcare services. Therefore, we recommend initiating interventions that advocate functional policy guidelines that will advocate for integrating palliative care within the available resources in primary care facilities to improve patients’ and their families’ health outcomes and quality of life.

## Supporting information

S1 Checklist COREQ_CHECKLIST. (PDF)

S2 Dataset Translated transcripts.
(PDF)
