# Supplementary material for: Struggles for provision of palliative care services at the primary healthcare setting in Tanzania; Experiences from health facility in-charges in Dar es Salaam; A qualitative study
Source: PLoS One. 2026 Feb 12;21(2):e0342485. doi: 10.1371/journal.pone.0342485 (PMC12900312; doi:10.1371/journal.pone.0342485)
Supplement: S2 Dataset — Translated transcripts. (PDF) [file pone.0342485.s002.pdf]

APPENDIX NO.1 TRANSCRIPTS IN ENGLISH LANGUAGE

HF-INCHARGE NO.1

I: Interviewer
R: Respondent

I: Thank you. Please tell us, in general, what services are provided at your facility?
R: Generally, most of the services provided here are outpatient services, although we also offer maternity services since pregnant women come here to give birth. After delivery, we keep them until they are in good condition before allowing them to go home. We provide all services to outpatients such as doctor consultation, laboratory tests, CTC services, and other services.

I: Thank you very much. I would also like to know what palliative care services are available at your facility.

R: *When you say palliative care, do you mean those diseases that are often not given much priority, or could you please explain it more?*

I: Palliative care refers to treatment for chronic or life-threatening diseases that cannot be cured in the same way we treat Malaria or Typhoid. Palliative care is for patients with Diabetes, Hypertension, etc.

R: *Okay. For those diseases, we provide services here because many patients come from their homes to receive initial care before being referred. Therefore, we serve patients with Diabetes, Hypertension, heart diseases, kidney diseases, etc. We identify them by listening to their symptoms and conducting preliminary tests, which can show if their blood pressure or blood sugar levels are high. We treat them, and if we find that referral is necessary, we refer them to higher facilities. We can also schedule follow-up appointments depending on the patient's condition — such as after one week, two weeks, or a month — as we monitor their medication use.*

I: On that note, I would like to know — for these chronic, non-communicable diseases and even communicable ones like HIV, which are truly life-threatening — what kind of palliative care do you provide?

R: *Indeed, we also provide general health education. For example, for an HIV patient, we educate them on how to maintain their health — including proper nutrition and using various protective measures. For a diabetic patient, we educate them to prevent other complications. A diabetic patient is taught how to take care of their health; for instance, if they develop a large wound and fail to treat it properly, it can even lead to disability. So, we give them both education and care.*

We have community health workers (CHWs) who provide home-based health services and education at the household level,. Therefore, if a patient needs home care, we connect them with these providers — they are based here at the facility and are assigned to different neighborhoods. We also collaborate with local government leaders, which helps us to know where each patient lives and how to reach them.

I: I see. You educate patients on eating nutritious foods. What happens if a patient cannot afford proper food? How do you handle such cases?

R: *Yes, such patients exist — those who are not financially stable. But by the time a patient reaches the hospital, we believe they have eaten something the previous day or that morning, which enabled them to get here. Therefore, we advise them to eat whatever foods are available in their area instead of thinking about foods that are unavailable locally.*

Some people actually have the right kinds of food but eat without a proper plan. For example, a child under five may come in with poor health, but when you ask the mother about food, she mentions all kinds of foods — peanuts, bananas, and many others. It turns out she simply doesn't plan meals well. You may find that she feeds the child only one type of food — for example, carbohydrates from morning to evening — while the child also needs vitamins and proteins.

So, we advise her that, for example, from the vegetables she cooks, she can give the child the soup. If she cooks beans, she can give the child the soup as well — it provides both nutrients and proteins instead of just giving ugali or potatoes alone. It may also happen that she has a papaya tree at home or can even ask a neighbor for some, without realizing she can give papaya to her child. Therefore, we advise them to use locally available foods for a balanced diet.

I: Have you ever had a client to whom you provided spiritual support? For example, someone who came to talk about their diabetes or hypertension—those life-threatening illnesses—do you offer them any kind of spiritual support?

R: *Yes, that's true, although everyone has their own beliefs. We often meet clients who hold certain beliefs about their illnesses, or who have been told something that affects their faith, and this can make their health deteriorate depending on what they believe. When they come to us, we sometimes realize that the problem is more about their belief than science. We talk to them, explaining that scientifically, things may be possible, but they can still pray to God according to their own faith — not by fasting excessively or wishing harm upon others. Some people are told things like "your uncle" or "your aunt" did something to cause their illness. We advise them that their condition may be caused by one, two, or three scientific reasons, so they should follow their faith positively — pray for good things, not destructive ones.*

I: Have you ever had a patient with cancer — once you diagnosed you find having cancer?

R: *Yes, it happens.*

I: As you know, cancer is a serious illness. How do you help such a patient?

R: *For such a patient, since we provide the initial care (first aid), we educate them and tell them that we will refer them for further tests because we don't have the equipment to confirm the diagnosis. We explain that based on certain signs and symptoms, there's a possibility it could be cancer, so we send them for a test to be sure. We also encourage them by saying that once you get tested and know the truth, it's the good way of knowing your health well perhaps you've had a wound for a long time, struggled here and there, spent money without answers — but once you get the test, if it turns out to be cancer, that will be your breakthrough, not the end. It will mark the end of the problem or the beginning of healing.*

I: I see — that's psychological support. Do you also have other means of managing pain or other symptoms? For example, cancer patients often experience severe pain — how do you manage that?

R: *We manage it according to the level of our facility. There are some medications that we are not authorized to prescribe, but we do help relieve pain as much as possible — for example, giving medication to help the patient sleep and rest better.*

I: Okay, thank you very much. Do you have any guidelines that you follow?

R: *Yes, we do have guidelines.*

I: Thank you very much for your good answers. Please tell us, how exactly are palliative care services provided at your facility?

R: *What do you say?*

I: I mean, what procedure do you follow when treating your palliative care patients?

R: *The procedure is as I mentioned earlier — the patient registers just like any other client. When they reach the consultation room, they explain how they feel (their medical history), or sometimes they may already have started treatment elsewhere, or they may be a new patient. Once we understand their condition, we often give them priority. For example, if someone comes in with severe hypertension, you can't make them wait in line. We also write a few key notes on their file so that whoever attends to them next can easily understand and provide quick assistance.*

I: Thank you very much. We've discussed many things in detail, but is there anything else you'd like to share based on our discussion?

R: *Of course. I'd like to mention that there are people who have studied palliative care as a special course — I believe they exist. I would really love if such trained professionals could be deployed to lower-level facilities like ours to support us in our daily work. For example, those trained in cancer management could work with us here on the ground — even just one of them would make a big difference. (There is a lack of palliative care professionals in our facilities.)*

I: Thank you very much. Our discussion has gone very well, and we really appreciate your time — we've learned a lot. I can see you have great experience in these matters — congratulations. We have finished our discussion, and I wish you all the best in your work.

HF-INCHARGE NO.8

I: We would like you to tell us what services are provided here at your facility.

R: *We provide outpatient clinic services as well as maternity services, meaning we handle pregnant women for antenatal clinics and normal deliveries, as well as cesarean sections. In the OPD we provide general outpatient services, eye services, dental services, tuberculosis services, HIV services, international vaccinations, hepatitis vaccinations, and family planning. We also provide voluntary HIV testing.*

I: Okay, thank you. You offer many good services, and I really appreciate that. I would like to know what palliative care services are available at your facility.

R: *Palliative care, honestly, we don't have (I: none). But we do attend to patients who have chronic illnesses. We have special clinics for diabetes and hypertension, but we cannot say that we offer palliative care as palliative care. However, when they come to the clinic, we give them their medications, do laboratory tests, and monitor them. Since we do not admit patients and we don't have specialist doctors here, whenever we find cases that are beyond our capacity, we refer them to Amana Hospital.*

I: Okay, thank you. Do specialist doctors sometimes come here?

R: *Yes, we have three pediatric specialists and one obstetrician-gynecologist. But we also work jointly with specialists from Amana Hospital to run our diabetes and hypertension clinics.*

I: Aha, okay. Thank you very much. Perhaps when those specialists come, we can assume that patients with hypertension receive both medication and counseling. So, that's still a form of palliative care, though it may not be formally included in your SOPs. Thank you very much for your good answers. Please tell me, as you mentioned, those services are provided generally but not in detail — is there anything else you would like to add to this short discussion, anything you'd like us to talk about together?

R: *Thank you. I would like to commend you for conducting this research. I think it would help if palliative care were included in the school curriculum — it should be a proper topic so that people can be taught about it, because not many people know what palliative care is.*

I: Do you think there is a need for it?

R: *There is a great need.*

I: Do you have a social worker here at the facility?

R: *Yes, we do. The social worker, as we know, is responsible for giving exemptions, which is one of the components of palliative care.*

I: So, after this, I'll ask you to direct me to the social worker so I can get some information from them.

R: *Okay. Thank you very much. I wish you a good morning, I know you have a lot to do and visitors as well. May God go before you. Thank you so much.*

HF-INCHARGE NO. 13

I: We are here this morning at Buguruni Health Centre, where we have been warmly welcomed by the facility in-charge. Good morning, Madam.

R: *Good morning.*

I: Thank you very much. We are here to conduct a study related to palliative care (PC). I would like to inform you that the interview will take only a few minutes. Your participation is voluntary, and you may withdraw at any time. Confidentiality of your information will be maintained. All data collected will be used for improving PC services in Ilala Municipality. If you have any questions, you may contact the principal investigator. Do you have any questions so far?

R: *No.*

I: Are you willing to continue with this interview?

R: *Yes, I agree to continue — no problem.*

I: Thank you very much. I'll start by asking your personal details. How old are you, Madam? … Very sorry madam, Now that we've finished the personal section, I would like us to proceed to the facility information, especially regarding palliative care. Please tell us, what general services are provided at your facility?

R: *We provide a variety of services — outpatient department (OPD), reproductive and child health (RCH), laboratory, radiology, pharmacy, dental and eye care, surgery, oral and dental services, CTC (Care and Treatment Clinic for HIV), emergency services, hazardous waste disposal, and pediatric services.*

R: *We have a total of 121 staff members: 88 are employed by the municipal director across different areas, 18 are part of the reserve team under MDH contracts, and 15 are temporary staff employed by the director to fill workforce gaps. These temporary workers serve in reception, pharmacy, nursing, and laundry. For example, in the pharmacy, we have one*

pharmacist and one pharmaceutical technician, who cannot work 24-hour shifts alone, so we hired additional support. The same applies to records and anesthesia — we have hired temporary staff to meet service demands.

I: Okay. Do you provide palliative care services here at your facility?

R: *Yes, we do provide for patients who need such care. For example, TB and HIV patients receive those services. However, we don't provide all types of palliative care because we lack facilities like an admission ward, although such patients exist in our community.*

I: You mentioned radiology services — do you offer cancer screening?

R: *Yes, we conduct cancer screening.*

I: Okay. We've seen that you have patients. Do you also have clinics for diabetes or hypertension?

R: *Yes, we do. We have specialist doctors who come here. For example, today we have a cardiologist who runs a special clinic, and on Fridays, we have an internal medicine specialist who is also an oncologist from Amana Hospital.*

I: That means patients with life-threatening diseases such as hypertension, diabetes, and mental illness have their own clinics. So definitely, palliative care is being offered, depending on the context. What types of palliative care services do you provide generally, for instance, to HIV patients and others?

R: *Of course, for HIV patients we provide care at the hospital level. We have a CTC department, and they have their own clinic days. They receive services here, and they can also be provided with home-based care.*

I: For those home-based services, do you have specific people who provide them?

R: *Yes.*

I: What kind of services do they provide at home?

R: *It depends on the patient's condition. If the patient cannot come to the hospital, they are provided with medicines, lab tests, and results at home. Usually, these patients have a health supporter who updates us if the patient is unable to reach the facility, and then the health worker and community volunteer visit them.*

I: I see. And such patients might be using medications, but he might still need more than just medication?

R: *Yes, most challenges are economic. We work with a particular group that provides that kind of support.*

I: So there's a specific group for that?

R: *Yes, they visit patients to assess their needs and determine what kind of social support they require — some provide food, others clothing. For example, if the patient was a parent and has children, the group may help support the children.*

I: How do you identify those who need that kind of support? Do you have a department that handles this?

R: *Yes, we have a social work department.*

I: I see — so the service is well-organized. Do you provide food for such patient and what about those patients who are emotionally distressed or have lost hope — how do you support them and their families?

R: *Of course, as you know, chronic patients need counseling all the time. So whenever they come, we counsel them. If we see that the patient needs more advanced medical support, we give them a referral to Amana Hospital. As a facility, we can help facilitate transport or sometimes request an ambulance to transfer them.*

I: Is there anything you'd like to add or suggest that could improve palliative care services, based on our discussion?

R: *First, we wish to have more specialist doctors visit our facility in different areas — pediatrics, internal medicine, and RCH. This would help reduce congestion and bring services closer to patients. Many of our clients are low-income earners, so when you refer them to Muhimbili Hospital, they see it as a burden and say they can't afford it. If they could get services here, it would be easier for both them and us and improves service.*

I: Thank you. Besides increasing the number of specialists, what else do you think could help improve the knowledge or skills of existing staff regarding palliative care?

R: *I think training is very important.*

I: Have you ever received any palliative care training here?

R: *No, we haven't.*

I: But are there people who studied it formally, like as a course in college?

R: *Yes, but we also share knowledge among ourselves. Every Wednesday we hold an SME, so if there's something new to learn, an update, or training feedback, it's shared there. We also discuss issues related to palliative care — like what improvements to make or what to implement.*

I: Thank you very much. I appreciate your time. I wish you all the best and a good day.

R: *Thank you very much.*

HF-INCHARGE NO. 15

I: Thank you very much. What kind of services do you provide here?

R: *We provide all hospital-related services such as OPD, IPD, delivery, surgery, and others.*

I: We would like to understand what palliative care services you offer.

R: *Yes, we do have a service for visiting patients at home, but I wouldn't call it palliative care. We don't have a specific palliative care program for patients — unless you clarify what exactly you mean. But for palliative management, no.*

I: Maybe to clarify a bit — how do you manage pain for the patients who come here?

R: *I think when you talk about palliation, it usually applies to a person with a chronic illness — and by the time you provide palliation, it means other management options have failed, so it's about ensuring the person spends their remaining time comfortably. So, in terms of pain management — no. We usually receive patients who are already under palliative care elsewhere, like from Ocean Road or Muhimbili National hospital. Some come while on oral morphine which is not available in our facility: others may be using local herbs. But we ourselves don't offer palliative management.*

We deal with other conditions — for example, if a patient comes with severe gastric pain, we give them medication to help with that, or if someone has a very painful leg, we provide immediate treatment. But long-term management — no.

I: Do you offer any psychological support for clients with long-term illnesses?

R: *That depends on the individual doctor. If the doctor feels the need to offer counseling, they do so. We receive many such patients. Currently, I have a patient with liver cirrhosis who is in the terminal stage, one with pancreatic cancer, another with CA prostate in terminal care, and one with a muscle-related disease who has also been classified as terminal. We usually just talk to the patient and their family — so it's more of a spontaneous counseling session depending on the doctor's time. Otherwise, we don't have a structured service for that.*

I: Do you offer any social support to such clients?

R: *Not really. Maybe in rare cases when we see that the family truly cannot afford care. In those individual cases, we might waive a wound dressing fee or consultation charge — but it's case by case, depending on the day and situation. There's no specific arrangement for social support.*

I: What about spiritual support?

R: *Umm… I can't say for sure, but we don't restrict it. For example, if a patient who has been admitted requests prayers or a visit from a religious person, we allow that — but we ourselves don't provide that service.*

I: Thank you very much. Would you say that palliative care services are needed?

R: *Palliative care services are highly needed. We really need them. I don't understand why Tanzania doesn't have such services. I studied in Uganda, and I remember back then there was a hospice. I've never heard of one in Tanzania — maybe they exist in some regions. These services are necessary not only for chronic illnesses but also for the elderly. They need end-of-life care. We all know when a patient has reached the final stage, and sometimes it's not even about the illness anymore — it's just part of life's process. So yes, I believe we need it.*

I: Thank you very much, Doctor. I made it brief as you requested.

HF-INCHARGE NO. 9

I: Thank you. Now we'll move on to the specific questions related to palliative care. I appreciate your readiness. Could you please tell me what general services are provided at your facility?

R: *The services provided here include reception, where patients are registered, then they go to see the doctor. After that, they may go to the laboratory, then to the pharmacy for medicines. If the patient needs admission, we admit them. We also offer maternity services — women come here to give birth. We conduct cesarean deliveries, and we have mother, father, and child clinics (RCH) as well as family planning services.*

I: So you have clinics for fathers, mothers, and children, and you perform surgeries for mothers during childbirth?

R: *Yes.*

I: Besides maternal care, are there other services?

R: *Do you mean surgical services?*

I: No — for example…

R: *We also provide services for people living with HIV/AIDS. We also offer services for TB patients, dental care, and eye care.*

I: Thank you. So we are continuing with the acting in-charge of the facility, who has explained the various services offered here, including dental and eye care. Now we'll proceed.

R: *Yes.*

I: Thank you very much. I'd like to understand what palliative care services are provided here.

R: *Okay. Palliative care here mainly applies to those who are screened for cervical cancer.*

I: Yes.

R: *Once they are diagnosed, they start receiving medication here — and beyond that, they receive palliative care.*

I: What other patients do you receive here, perhaps those who attend regular clinics?

R: *Mostly patients from the CTC — those living with HIV/AIDS.*

I: Do they receive palliative care?

R: *Yes.*

I: Palliative care, as we know, is for patients with chronic, life-threatening diseases — conditions without complete cure, unlike those with short-term treatment, you tell them to use medics may be for seven days, but the other diseases they must come to visit daily, and you insist them to use everyday medications, they are already in life threating diseases, So, do you have patients who take lifelong medication of whom they use these medications daily until the end of there lives?

R: *Yes, we do. Those living with HIV, hypertension, and diabetes come here, and we provide them with medication that we have in stock.*

I: Okay. So what kind of palliative care services do you provide to these patients — for example, those with diabetes, hypertension, or HIV?

R: *We have special clinics for them. When they come, there must be a doctor present. The doctor speaks with them, gives advice, and provides their medicine. So the main services we offer are counseling and medication.*

I: Okay.

R: *The counseling involves teaching them how to live with their disease, how to properly take their lifelong medication, and ensuring they don't skip doses. So every time they visit, they receive continuous counseling.*

I: So that means you provide psychological support. Do you ever get cancer patients in critical condition — for example, cervical cancer patients experiencing severe pain?

R: *Hmm… we have never meet patients in extreme pain. There was only one elderly woman I met who had breast cancer from another place. She only came to clean the wound, She had a large ulcerated wound, very painful to the point of near shock. That's the only such case I've seen since I came here.*

I: How did you manage that pain and shock?
R: *We managed the shock through resuscitation, infusion, and wound dressing. After that, we referred her to Amana Hospital for further treatment since we couldn't provide adequate care here. She understood, but due to financial challenges, she couldn't go immediately. Later, her family managed to take her, and when she came back to us, she was doing much better, we opened the wound to see its progress, the wound had improved significantly.*

I: Okay, so you referred her and managed only the initial stage?

R: *Yes, exactly.*

I: Do you have any pain medications for patients in severe pain?

R: *Only basic painkillers — we usually have diclopar, diclofenac, and paracetamol. Those are what we use for pain management.*

I: Okay. What about social support services?

R: *Yes, we offer social services.*

I: Oh, you do? What kind?

R: *Usually, if a patient has a social problem — for instance, financial difficulties or lack of support — they meet with the social welfare officer. If necessary, we verify their story to confirm if they truly lack support or relatives, and then continue helping them accordingly.*
I: So you must get approval from them, and may be another patient comes, and basing on her situation, you advice on getting spiritual support, like to that extent of the disease, God can do everything.
R: *Telling a patient who is at a critical situation.*
I: Yes, one of the advices is on spiritual support?
R: *Yes, as nurses, we often encourage patients — give them hope — but we don't have a specific person assigned to provide spiritual support.*

I: Thank you very much for your good answers. Could you tell us how palliative care services are provided in your facility?

R: *They are provided mainly through the clinics. Each group of patients with life-threatening illnesses has its own clinic where they receive their services.*

I: So each group has its own clinic and care sessions. Thank you. Is there anything you'd like to add or share regarding palliative care? We have discussed so many issues.

R: *I think spiritual care is an important area. It would be helpful if we could have someone responsible for that — it would help patients live with hope because faith plays a big role in healing and comfort.*

I: So in terms of psychological support, are the counselors doing well?

R: *Yes, we have counselors — they are the ones who meet the patients and provide counseling.*

I: Do you have guidelines for that — for counseling or palliative care in general?

R: *Yes, we have palliative care guidelines, and health professionals review them regularly to ensure they know what needs to be done.*

I: Excellent. We're grateful for your insights. The information you've shared will help policymakers improve palliative care services in our city and across Tanzania.

R: *We'll be very grateful if that happens.*

I: It will happen. I wish you all the best in your work. Thank you very much.
